# Reduction of the uncertainty of flood projection under a future climate by focusing on similarities among multiple SSP-RCP scenarios

**DOI:** 10.1038/s41598-025-16327-4

**Published:** 2025-09-22

**Authors:** Y. Kimura, Y. Hirabayashi, D. Yamazaki

**Affiliations:** 1 Data Analytics Department, MS&AD InterRisk Research & Consulting, Inc., 2-105, Kanda Awajicho, Chiyoda-ku, Tokyo, 101-0063 Japan; 2 Institute of Industrial Science, The University of Tokyo, 4-6-1 Komaba, Meguro-ku, Tokyo, 153-8505 Japan; 3 Department of Civil Engineering, Shibaura Institute of Technology, 3-7-5 Toyosu, Koto-ku, Tokyo, 135-8548 Japan

**Keywords:** Hydrology, Climate change

## Abstract

Climate projections contain the uncertainty due to the internal variability of the climate system, including its chaotic nature. While the uncertainty due to the internal variability can be theoretically mitigated by executing large ensemble simulations with perturbed initial conditions, only a limited number of large-ensemble experiments are available in CMIP6 future scenario dataset. Here we propose a method that increases the effective ensemble sampling size in evaluations of future projection by integrating multiple SSP-RCPs for a period corresponding to a specific increase in temperature from the preindustrial level (i.e., X°C warming). The success of the method was assessed by investigating whether the uncertainty due to small number of ensemble members could be reasonably reduced. First, we confirmed that the spatial distributions of the future flood magnitude change were similar under a 2 °C warming in all SSP-RCP scenarios. Additionally, the uncertainty due to the different SSP-RCPs (5–10%) was smaller than the differences between different warming levels such as between 2 and 3 °C (around 20–50%), suggesting differences among SSP-RCPs as to future flood discharge change are relatively small. These results suggested that integrating SSP-RCPs to increase the effective ensemble size was a reasonable approach, reducing unbiased variance among GCMs in about 70% of land grid points comparing to the result using SSP5-RCP8.5 alone.

## Introduction

Climate model outputs contain uncertainties arising from the internal variability of the climate system, model responses, and radiative forcing scenarios. Previous study showed that the average of multiple general circulation models (GCMs) has been found to align more closely with various observational data than the results of any single model^1^. Averaging multi-model outputs is expected to offset specific limitations of individual GCMs, such as challenges in representing complex processes and parameterizations, and to help achieve reasonable climate fields, as all climate models are designed to replicate the same Earth climate system in various aspects.

In addition to the multi-model approach, the ensemble simulation approach using multiple simulations with perturbed initial conditions is widely used to reduce uncertainty due to the internal variability of the climate system, including its chaotic nature (hereafter termed as “internal variability” in this paper). Lehner et al.^2^ demonstrated that internal variability poses a significant challenge in future climate change assessments, especially during the early decades or at lower levels of warming, where it can dominate over scenario-related differences on a global scale. The spatial distribution of hazards related to factors such as precipitation varies greatly due to internal variability^3–5^ and influences climate impact projections^6^. A large set of ensemble experiments, such as 50–100 runs conducted with a single GCM with perturbed initial conditions, demonstrates the magnitude of internal variability and shows that increasing the number of ensembles helps reduce it^7^. For example^8^, showed that, for precipitation and temperature, increasing the ensemble size leads to greater agreement (convergence) in projection results. Based on a large-ensemble climate simulation of a single GCM^9,10^, demonstrated that a small number of ensembles can lead to significant uncertainty, particularly for severe rainfall and severe floods.

However, studies that have used the output of GCMs in Coupled Model Intercomparison Project Phase 6 (CMIP6,^11^) under Scenario Model Intercomparison Project (ScenarioMIP, O’Neill et al.^12^) face limitations in addressing uncertainties in future projections because there are few GCMs that include large ensembles. Some studies that have used CMIP5 or 6^13–15^ to project flooding have focused on specific time periods, such as the end of the twenty-first century, but the lack of ensembles inevitably leads to large uncertainties. For example, Hirabayshi et al.^15^ examined future flood risk and associated uncertainty, but the variability in projections among GCMs was still large.

To address this problem, here we proposed a method that increases the effective ensemble sample size in evaluations of future projections, by integrating multiple SSP-RCPs over the period of a specific temperature rise from preindustrial level (i.e., X°C warming). Because each SSP-RCP has a different range of temperature rise for use in risk assessments for specific time periods, such as the end of the twenty-first century, different SSP-RCPs should be treated as different sampling pools. If the spatial patterns of changes at X°C warming can be considered similar among SSP-RCPs, then the different SSP-RCP scenarios can be considered to make up one sample pool to increase effective ensemble number (hereafter, “effective ensemble number” denotes the total number of ensemble members treated as a single pool by combining multiple SSP-RCP scenario simulations). This idea stems from past findings that show the same level of warming produces similar changes in climate variables. It is widely recognized that the accumulation of anthropogenic radiative forcing since 1850 exhibits a linear relationship with global warming (e.g., IPCC, 2021). In addition, similar linear or direct effect of the warming temperature on several climate-related variables have been reported. For example, Shiogama et al.^8^ demonstrated a linear increase in global mean precipitation with global warming across all SSP-RCP scenarios, except for SSP3-RCP7.0, which incorporates a unique aerosol scenario compared to the others. Hirabayashi et al.^16^ demonstrated that the projected increase in flood exposure across all GCMs and RCPs showed a strong correlation with rising temperatures.

This study investigated whether the projection uncertainty due to the internal variability could be reduced by increasing effective ensemble size through merging multiple SSPs and extracting periods with the same warming levels under each SSP. Precipitation, particularly daily maximum precipitation, is a major source of uncertainty in climate change projections^8^, such that flood projections are subject to even greater uncertainty. Thus, we focused on flood projection, specifically river discharge, and investigated the uncertainty of GCMs. First, we determined whether the distribution of changes in flood projections among different SSP-RCPs is similar and thus whether integrating SSP-RCPs to increase the effective ensemble size is justified, then we quantified the extent to which uncertainty can be reduced by our proposed method, which integrates SSP-RCPs.

## Data and methods

We execute global river hydrodynamics model simulations using runoff data from CMIP6 GCMs to discuss how to handle climate model ensembles to reduce the uncertainty due to internal variability. In particular, we investigate whether this uncertainty—often amplified by the limited number of ensemble members available for future climate simulations—can be mitigated by combining outputs from multiple SSP-RCP scenarios. The central idea is to extract the period corresponding to a specific global warming level (e.g., 2 °C above preindustrial) from each SSP-RCP scenario. If the spatial patterns of flood change are consistent across scenarios at the same warming level, these outputs can be integrated as a unified ensemble, thereby increasing the effective sample size without requiring additional simulations.

**Fig. 1:**
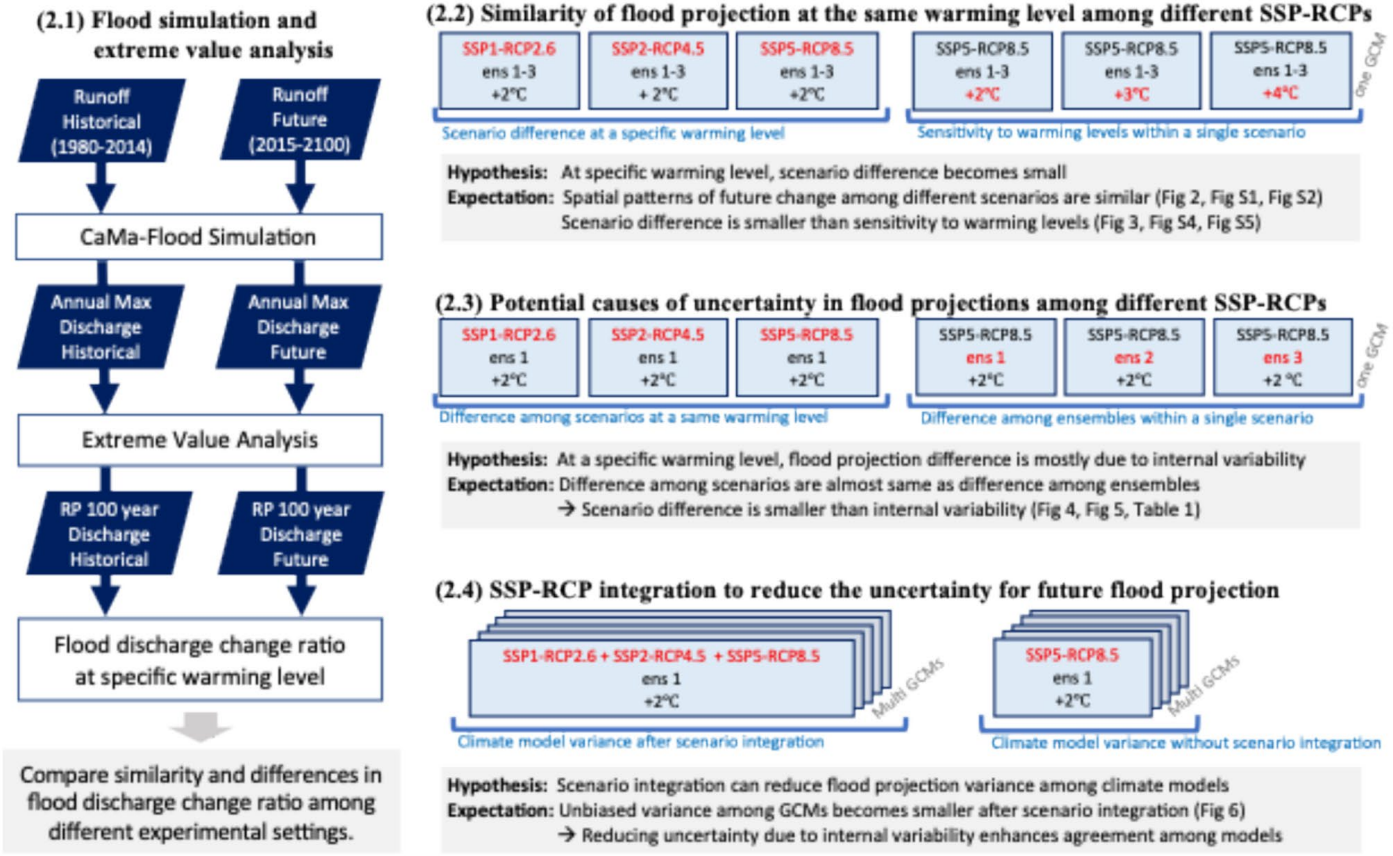
Simulation procedure flowchart and experimental settings. Each experimental setting shows representative combinations of SSP-RCP scenarios, ensemble members (ens), and specific warming levels (+ 2 to + 4 °C). Note that only illustrative combinations are displayed; the full range is analyzed in the corresponding sections.

The GCMs and the river model used in this study are described in Section “Flood simulation and runoff data”. To evaluate the validity of this approach, we first assess the similarity of flood projections among different SSP-RCPs at the same warming level by comparing spatial distributions and statistical characteristics of flood discharge change (Section “Similarity of the flood projection under the same warming level among different SSP-RCPs”). We then identify potential sources of uncertainty among scenarios (Section “Potential causes of uncertainty in flood projections among different SSP-RCPs”), and quantify the extent to which our proposed integration method reduces inter-model variance (Section “Ability of SSP-RCP integration to reduce the uncertainty for flood projection”). The calculation flow and experimental settings are summarized in (Fig. 1). Note that we primary focused on flood discharge change in this study, but the same method can be applicable to other flood-related variables such as water depth or inundation extent.

### Flood simulation and runoff data

A global river hydrodynamics model CaMa-Flood ver. 4.10^17,18^ was used to simulate river discharge with runoff from GCMs as input forcing. Runoff refers to the amount of water reaching to a river channel from land calculated in each GCM’s land surface process, and CaMa-Flood calculates how water moves along river networks. CaMa-Flood allows for the simultaneous simulation of river discharge, water depth, and flood inundation extent by representing floodplain topography at sub-grid scale. These outputs form the basis not only for evaluating changes in hydrological variables but also for constructing spatially explicit flood hazard maps^19^. In our study, these capabilities were leveraged to assess both the changes in discharge and their implications for inundation extent and associated flood risk under warming scenarios. For the CaMa-Flood model, consistency with historical river level, flow, and inundation area data has been demonstrated^20^. Additionally, uncertainty among models and scenarios for future projections has been examined^15,16,19^. A detailed description of CaMa-Flood is provided in^17,21,22^).

In this study, 6-arcmin resolution river simulations were forced with the daily runoff outputs of the GCMs for two time periods: historical (1980–2014) and future (2015–2100). For the latter, three scenarios based on a combination of SSP-RCPs (SSP1-RCP2.6, SSP2-RCP4.5, and SSP5-RCP8.5) were used. GCM-output runoff was converted from its original spatial resolution to 30-arcmin resolution through bilinear interpolation. Nine GCMs from independent institutes were used^15^: MIROC6, IPSL-CM6A-LR, GFDL-CM4, NorESM2-MM, ACCESS-CM2, INM-CM5-0, MPI-ESM1-2-HR, MRI-ESM2-0, EC-Earth3.

### Similarity of the flood projection under the same warming level among different SSP-RCPs

We first hypothesized that scenario difference on flood projection becomes small at a specific warming level. To proof this hypothesis, the similarity in the future flood discharge among the different SSP-RCP was investigated by comparing the spatial distribution of the flood discharge change ratio for each SSP-RCP. The flood discharge change ratio is calculated at 6-arcmin resolution and the flood discharge is the summation of the river channel and floodplain flow. The future flood discharge at specific warming levels (SWLs) of X°C above the preindustrial temperature was calculated as follows. (1) As in previous research^23^, SWLs were calculated as the year each SWL first surpassed a reference temperature relative to the preindustrial period (1850–1900), using a running mean of the 30-year global averaged annual mean temperature (Supplementary Tables S1). We selected the 30-year window centered at the SWL year with reference to previous studies(e.g.^23^). (2) Next, as to each catchment, we fitted the Gumbel distribution to the annual maximum discharge of the 30-year used for calculating at SWLs (30-year sample including 15 years before and 14 years after the SWL year) with the L-Moments method^24^. (3) Then, 100-years return period discharge for each grid point was calculated from the Gumbel distribution.

We used multiple ensemble simulations to increase the sample size for this extreme value calculation. Specifically, three ensembles were used in one SSP-RCP (Supplementary Tables S2), and the presence of extreme values was analyzed for 90 samples (30 years × 3 ensembles), by fitting to the Gumbel distribution. The same procedure was used to calculate the flood discharge for the historical period (1980–2014), and then the flood discharge change ratio was calculated using Eq. (1):

1 $$ChangeRatio= \frac{100yearDischarge\left(at X^\circ C\right) - 100yearDischarge\left(historical\right)}{100yearDischarge\left(historical\right)}$$

This ratio reflects the relative change in extreme flood magnitudes due to climate warming.

Even for the same GCM and under the same SSP-RCP, the timing of X°C warming may differ between different ensembles, due to internal variability. The timing of the 2.0 °C warming was therefore investigated in three multiple ensembles of IPSL-CM6A-LR under the same SSP-RCP, which showed a difference of only about 1 year between ensembles. The same was true for other GCMs (ACCESS-CM2 and EC-Earth3) with multiple ensemble experiments under the same SSP-RCP. Those results support the use of the same period for the other ensembles, assuming that the timing of the X°C warming is the same as “r1i1p1f1”, which is the first available initial condition ensemble member.

Whether the change trends in each grid are similar across various SSP-RCP scenarios was determined in a global-scale analysis, based on^25^. That study compared the differences in runoff, discharge, and related factors between the high-end climate scenario (RCP 8.5) and lower RCPs (RCP2.6 and RCP4.5). Then the flood discharge change ratios between the lower SSP-RCPs and the higher SSP-RCP were compared^25^. Also compared the differences in impacts between different warming levels, by plotting the average change attributed to one warming level (e.g., 1.5 °C) combined with another level (e.g., 2 °C) for each grid point on a scatter plot. In this study, we made a scatter heatmap plot to quantitatively evaluate the similarities and differences of estimated flood discharge change ratio between two simulations using different SSP-RCPs input. Here, the flood discharge change ratio of one simulation is compared to another simulation at each grid.

### Potential causes of uncertainty in flood projections among different SSP-RCPs

We then hypothesized that the flood projections differences among different scenarios at a specific warming level is mostly due to the internal variability, rather than the differences due to scenario-specific characteristics. To proof this hypothesis, the potential causes of uncertainty among the different SSP-RCPs with respect to the flood change ratio in the future climate was analyzed by comparing the spatial distribution patterns of the variation (standard deviation) of the flood change ratio among different ensembles (same GCM under SSP5-RCP8.5) and among different SSP-RCPs (same GCM). If the spatial distribution of variation among different SSP-RCPs of the same GCM was similar to that among the different ensembles (which is most likely due to internal variability), the differences due to scenario-specific characteristics can be said to be not so important in terms of the physical changes in flood magnitude. In that case, the SSP-RCPs could be treated as effective ensemble members during the time of X°C warming and thus merged to reduce the uncertainty due to internal variability.

In addition, we made a scatter heatmap plot to quantitatively evaluate the similarities and differences of estimated flood discharge change ratio among different three ensembles (same GCM under SSP5-RCP8.5) and among different three SSP-RCPs (same GCM and different ensembles). Then, we evaluated the difference and similarities of the two simulations by calculating Mean Absolute Error and Pearson Correlation Coefficient. These metrics are calculated for the three possible combinations of two simulations. We calculated the metrics for three different climate models (ACCESS, EC-Earth, and IPSL) and at 1.5 and 2.0 °C warming levels.

### Ability of SSP-RCP integration to reduce the uncertainty for flood projection

Lastly, we hypothesized that scenario integration can reduce flood projection difference among multiple climate models by mitigating uncertainties due to internal variability. To proof this hypothesis, the extent to which integration of the SSP-RCPs could reduce the uncertainty was quantified by comparing the variance among GCMs with respect to the change in the flood discharge during the historical climate to in response to 2 °C warming, first using only SSP5-RCP8.5 and then using our proposed method integrating multiple SSP-RCPs. Unbiased variance is an indicator that allows a comparison of the variance of data derived from different numbers of samples: a lower value indicates reduced uncertainty. In this study, unbiased variance was applied because sample sizes can vary depending on the method used to integrate the SSPs and ensemble members (see Supplement Text S2). Warming of 2 °C was chosen because it is used in many GCMs and SSP-RCPs.

Our proposed method integrates multiple SSP-RCP into a 90-year (or 60-year) time-series data pool at each GCM and then performs an extreme values analysis. The procedure involves the following: (1) A survey of the nine GCMs and three SSP-RCPs to identify those that reach 2.0 °C (Supplementary Table S1), which in this study yielded 21 GCM-SSP-RCPs. (2) Calculation of the flood discharge using multiple SSP-RCPs in one GCM and increasing the sample size (i.e., number of years) to mitigate the uncertainty due to extreme values. Specifically, three (or two) SSP-RCPs were used in one GCM, and an extreme value analysis was performed for 90-year (or 60-year) time-series data; flood discharge under the future climate was calculated in each GCM. For example, in the case of three SSP-RCPs in one GCM, the extreme value analysis performed for the annual maximum discharge for 90 years = 30 years × 3 SSP-RCPs). (3) Performing an extreme value analysis for the annual maximum discharge during the historical climate (1980–2014) according to each GCM to calculate the flood discharge and flood change ratio at 2 °C warming for each GCM using Eq. (1). (4) Calculation of the unbiased variance of the flood change ratio among the nine GCMs at each grid point.

## Results

### Similarity in the flood projection at the same warming level among SSP-RCPs

**Fig. 2:**
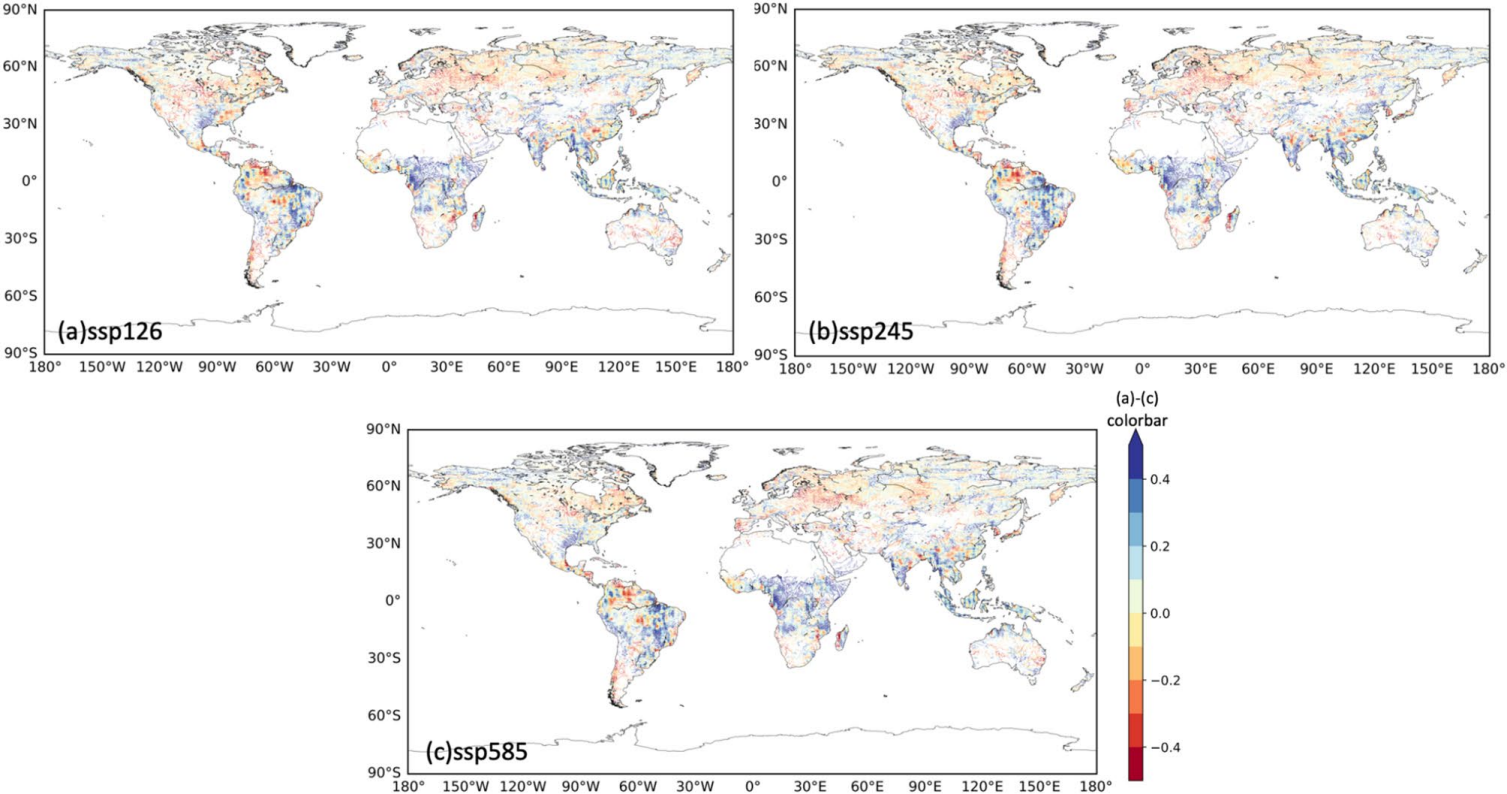
Spatial distribution of the flood change ratio from the historical climate to 2 °C warming. (**a**) SSP1-RCP2.6, (**b**) SSP2-RCP4.5, (**c**) SSP5-RCP8.5. Grid cells with a flood discharge in the historical climate < 100 m3/s were excluded. The results of IPSL-CM6A-LR GCM are shown here, while same results for other GCMs are in supplement.

The spatial distribution of the flood discharge change ratio from the historical climate to 2 °C warming in IPSL-CM6A-LR showed similar patterns among the different SSP-RCPs (Fig. 2). In all of them, flooding increased in many areas in Southeast Asia and low-latitude Africa, while it decreased from northern and Eastern Europe to western Russia, in central North America, and in northern South America. Thus, the differences among SSP-RCP scenarios were relatively small in comparisons of the flood change at a specific degree of global warming. The same analyses for EC-Earth3 and ACCESS-CM2 showed similar results (Supplementary Figs. S1, S2). The close-up views for Europe and Central Africa are shown in Supplementary Fig S3.

**Fig. 3:**
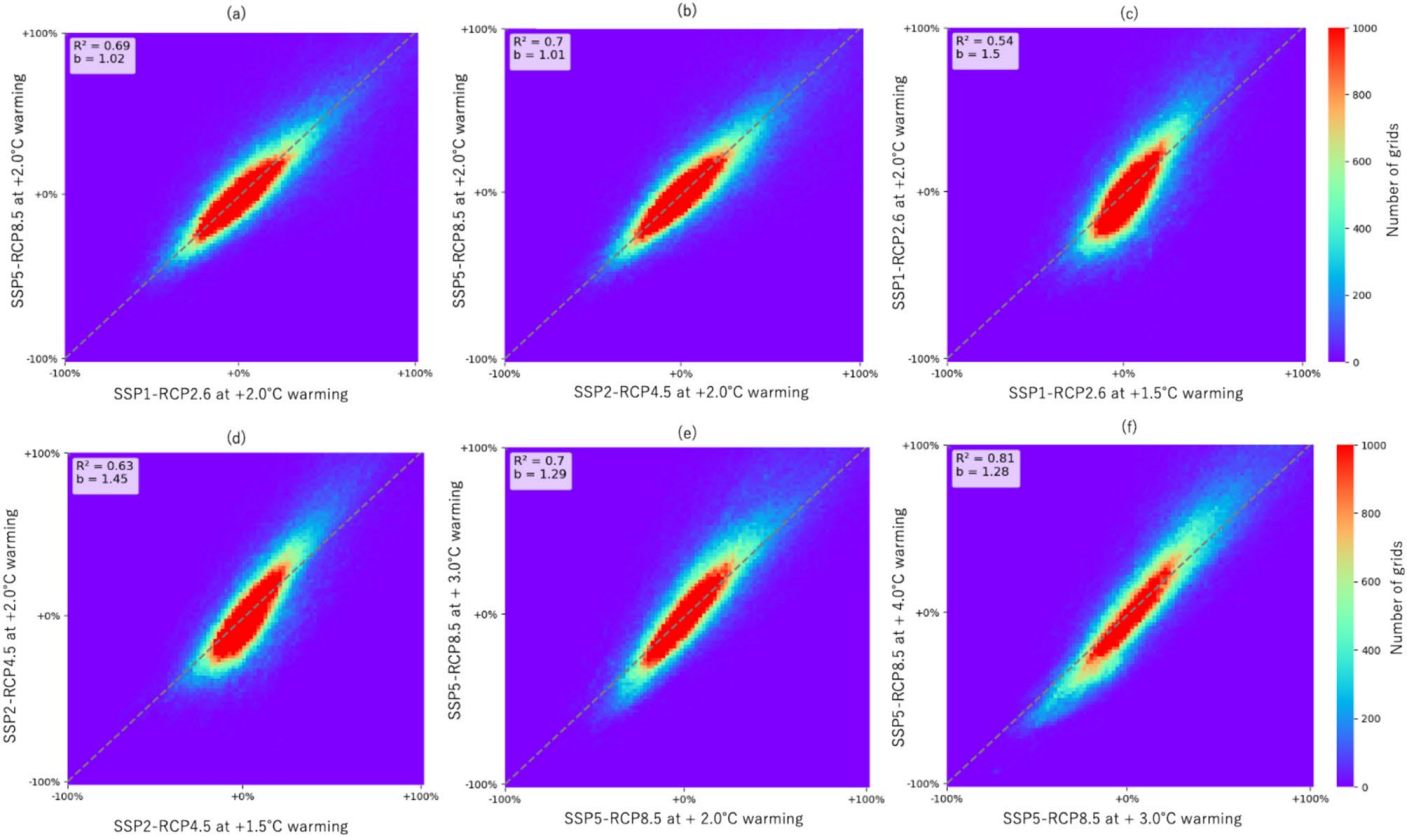
Comparison of the flood change ratio among two simulations. (**a**,**b**) Among two future scenarios characterized by the same warming level but under different RCP-SSP scenarios: (**a**) 2.0 °C warming between SSP1-RCP2.6 and SSP5-RCP8.5; (**b**) 2.0 °C warming between SSP2-RCP4.5 and SSP5-RCP8.5 (IPSL-CM6-LR). (**c**–**f**) Among two future scenarios at different warming levels: (**c**) 1.5 °C and 2.0 °C under SSP1-RCP2.6; (**d**) 1.5 °C and 2.0 °C under SSP2-RCP4.5; (**e**) 2.0 and 3.0 °C under SSP5-RCP8.5; (**f**) 3.0 and 4.0 °C under SSP5-RCP8.5. The dashed line is a 1:1 linear. “b” is the slope of the fitted line using principal component regression and the R value is the correlation of determination. The results of IPSL-CM6A-LR GCM are shown here, while same results for other GCMs are in supplement.

In the quantitative analysis, conducted at a global scale, scatter heatmap plot were used to diagram whether the trend of a change in each grid was similar between different SSP-RCP scenarios. Figure 3a,b show the flood change rate at the same warming level obtained from different SSP-RCPs in IPSL-CM6A-LR. A lot of grids are located around y = x, indicating that flood discharge is similar at the same warming level regardless of the SSP-RCP, the differences between y = x and the slope “b” of the fitted line using principal component regression is less than 5%. Figure 3c–f shows the flood change ratio for the different warming levels in IPSL-CM6-LR. For all SSP-RCPs, there was a tendency for increased floods with warmer temperatures. For example, the flood change ratio in most of the grid cells is larger for 3.0 than for 2.0 and the slope “b” of the fitted line of the flood change ratio was 1.29. The value of “b” was similar for 1.5 and 2.0 whereas for 3.0 and 4.0 the magnitude of the difference in the flood change ratio between different warming levels was 20–50%, which was larger than the difference among different SSP-RCPs at the same warming level. A comparison of the scatter heat maps for ACCESS-CM2 and EC-Earth3 is shown in Supplementary Figs. S4 and S5, which summarizes Table S3. For EC-Earth3 and ACCESS-CM2, the trend was the same as in (Fig. 3). We also confirmed that the spatial distributions of the future flood magnitude change were similar under a 2 °C warming in all SSP-RCP scenarios in case of EC-Earth3 and ACCESS-CM2. Additionally, the uncertainty due to the different SSP-RCPs (5–10%) was smaller than the differences between different warming levels such as between 2 and 3 °C (around 30–50%), suggesting differences among SSP-RCPs as to future flood discharge change are relatively small in both EC-Earth3 and ACCESS-CM2.

The above results showed that future flood changes, such as flood discharge, differ between different SSP-RCPs, but the differences are smaller than those between different warming levels. Although trends may vary by GCM, integrating different SSP-RCPs may reduce the uncertainty due to small number of ensemble members. In the next section, we analyzed the causes of uncertainty among different SSP-RCPs in the flood discharge of a future climate, by examining the spatial distributions of the standard deviations.

### Potential causes of uncertainty in flood projections among SSP-RCPs

**Fig. 4:**
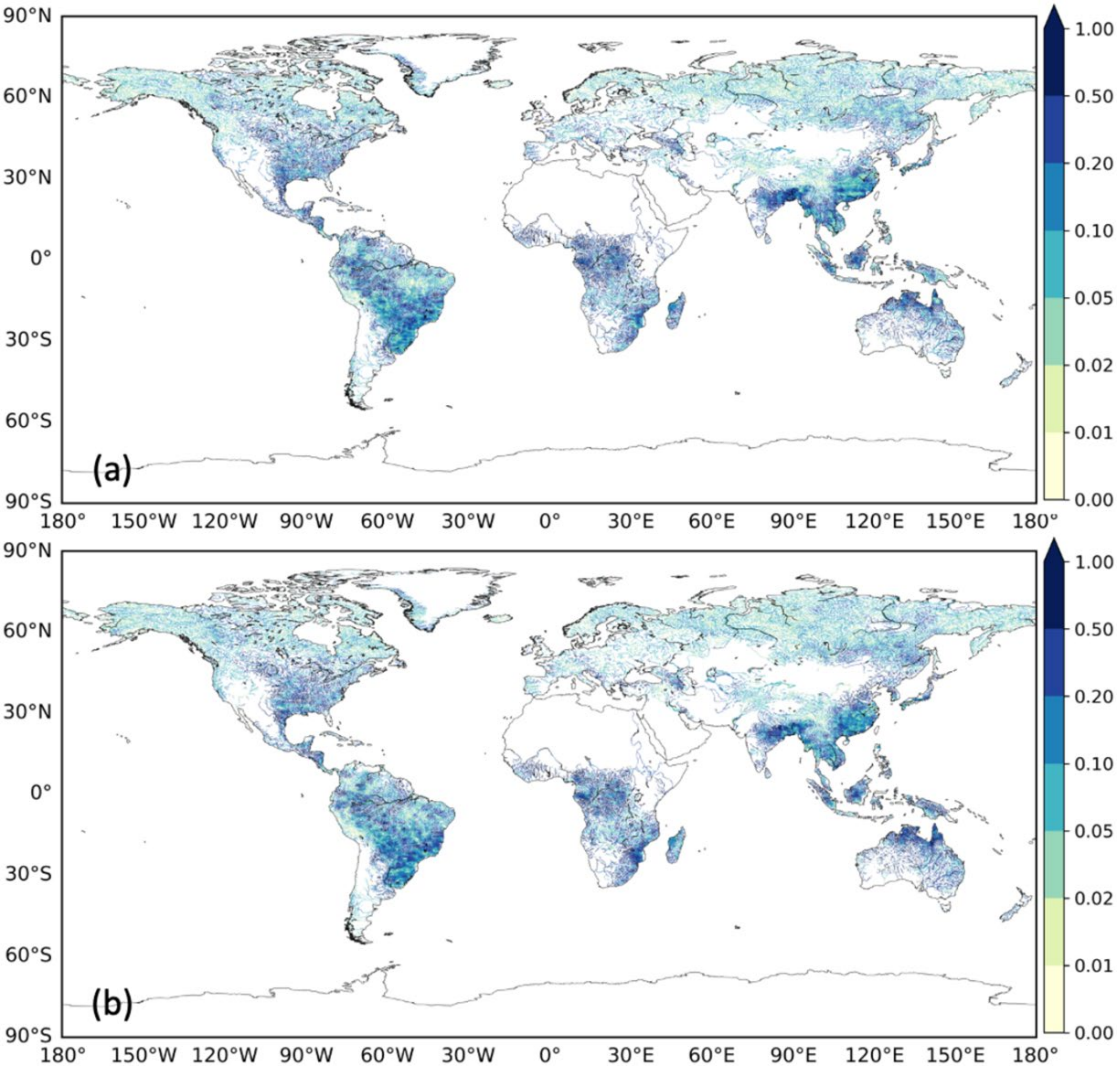
Spatial distribution of the standard deviation of the flood change ratio from the historical climate to 2.0 °C warming. (**a**) Standard deviation among three different SSP-RCPs; (**b**) standard deviation among three different ensembles (SSP5-RCP8.5). Grid cells with a 100-year RP discharge in the historical climate < 100 m3/s were excluded. The results of IPSL-CM6A-LR GCM are shown here.

To investigate the causes of uncertainty among different SSP-RCPs with respect to flood discharge in a future climate, the global spatial distribution of the variability (standard deviation) in the flood change ratio from the historical climate to 2.0 °C warming was compared among different SSP-RCPs of the same GCM and among different ensembles of the same GCM under SSP5-RCP8.5 (Fig. 4). Figure 4a shows the large variability and thus the large uncertainty in the same regions, including the Mississippi River (USA), the low-latitude region of Africa, and the region extending from China across Southeast Asia. The close-up views for Europe and Central Africa are shown in Supplementary Fig S6.

The main source of the variation among different ensembles of the same GCM under the same SSP-RCP is internal variability. This variation had a similar spatial distribution as in Fig. 4b, which implies that the main source of the difference among SSP-RCPs was internal variability. Thus, the differences in the pathways of warming to the same temperature increase can be said to be not very large, in terms of the physical changes in flood magnitude under the spatiotemporal resolution covered in this study.

**Fig. 5:**
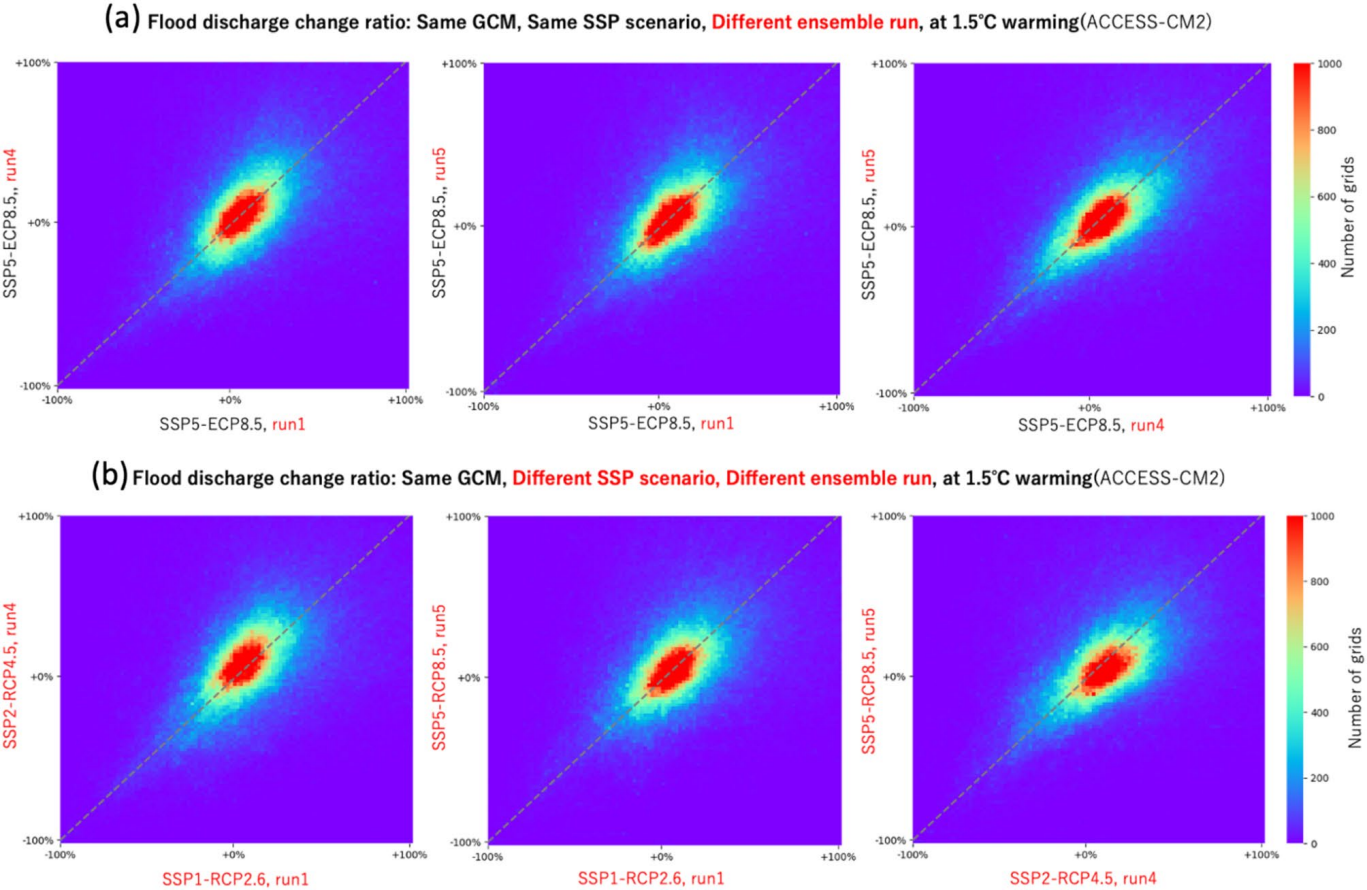
Scatter heatmap plot showing the similarities and differences of estimated flood discharge change ratio between two simulations. (**a**) using different SSP scenarios. (**b**) using different ensemble run input. The results of ACCESS-CM2 GCM are shown.

In the top panels of Fig. 5, the flood discharge change ratios of the simulations with “Same SSP scenario, different ensemble runs” are compared (note: same model at 1.5 °C warming). Thus, differences of two simulations are due to the internal variability. Then, in the bottom panel of Fig. 5, we compared the flood discharge change ratio between the simulations with “different SSP scenarios, different ensemble runs”. Thus, the difference in the bottom panel is considered to be due to both “internal variability and scenario difference”.

Then, we evaluated the difference and similarities of the two simulations by calculating Mean Absolute Error and Pearson Correlation Coefficient as shown in (Table 1). These metrics are calculated for the three possible combinations of two simulations (i.e., three panels in top of Fig. 5), and the mean and standard deviation of these metrics are listed in (Table 1). We calculated the metrics for three different climate models (ACCESS-CM2, EC-Earth3, and IPSL-CM6A-LR) and at 1.5 and 2.0 °C warming levels.

**Table 1:** Quantitative evaluation of similarities between ensembles. (Mean ± standard deviation of MAE between 3SSP or 3 ensemble).

	Mean absolute error (MAE)			Pearson correlation coefficient		
Climate model	IPSL-CM6A-LR	EC-Earth3	ACCESS-CM2	IPSL-CM6A-LR	EC-Earth3	ACCESS-CM2
Comparison setting						
SSP5-RCP8.5, different ensemble runs, 1.5℃	0.15 ± 0.03	0.15 ± 0.02	0.18 ± 0.00	0.50 ± 0.18	0.58 ± 0.11	0.52 ± 0.03
different SSPs, different ensemble runs, 1.5℃	0.15 ± 0.03	0.14 ± 0.02	0.19 ± 0.00	0.50 ± 0.19	0.61 ± 0.10	0.49 ± 0.02
SSP5-RCP8.5, different ensemble runs, 2.0℃	0.22 ± 0.05	0.16 ± 0.02	0.20 ± 0.00	0.48 ± 0.23	0.65 ± 0.10	0.57 ± 0.02
different SSPs, different ensemble runs, 2.0℃	0.21 ± 0.05	0.16 ± 0.02	0.20 ± 0.00	0.49 ± 0.24	0.65 ± 0.09	0.58 ± 0.02
different SSPs, different ensemble runs, 1.5℃vs2.0℃	0.22 ± 0.03	0.17 ± 0.02	0.22 ± 0.01	0.40 ± 0.21	0.60 ± 0.07	0.49 ± 0.04
different SSPs, different ensemble runs, 1.5℃vs3.0℃	0.25 ± 0.02	0.22 ± 0.01	0.27 ± 0.02	0.38 ± 0.13	0.57 ± 0.04	0.42 ± 0.05

We found out that the Mean Absolute Errors are almost the same between “comparison of same SSP and different ensemble runs” and “comparison of different SSP and different ensemble runs”. This indicates that the uncertainties due to scenarios are almost negligible compared to the uncertainties due to internal variability, at least at 1.5 and 2.0 °C warming level. The results for Pearson Correlation Coefficient suggested the same pattern. These results support our idea of integrating different SSP simulations to increase effective ensemble size, given that the differences are mostly due to internal variability, even in different SSP simulations, when the warming level is the same. We also did the same analysis for the comparison of different warming levels under the same RCP scenario, and found that the difference due to the warming level is larger than the uncertainties due to internal variability, for all climate models.

As noted above, integrating different SSP-RCPs may reduce the uncertainty due to small number of ensemble members.

### Reduction of uncertainty in flood projection by the proposed method

**Fig. 6:**
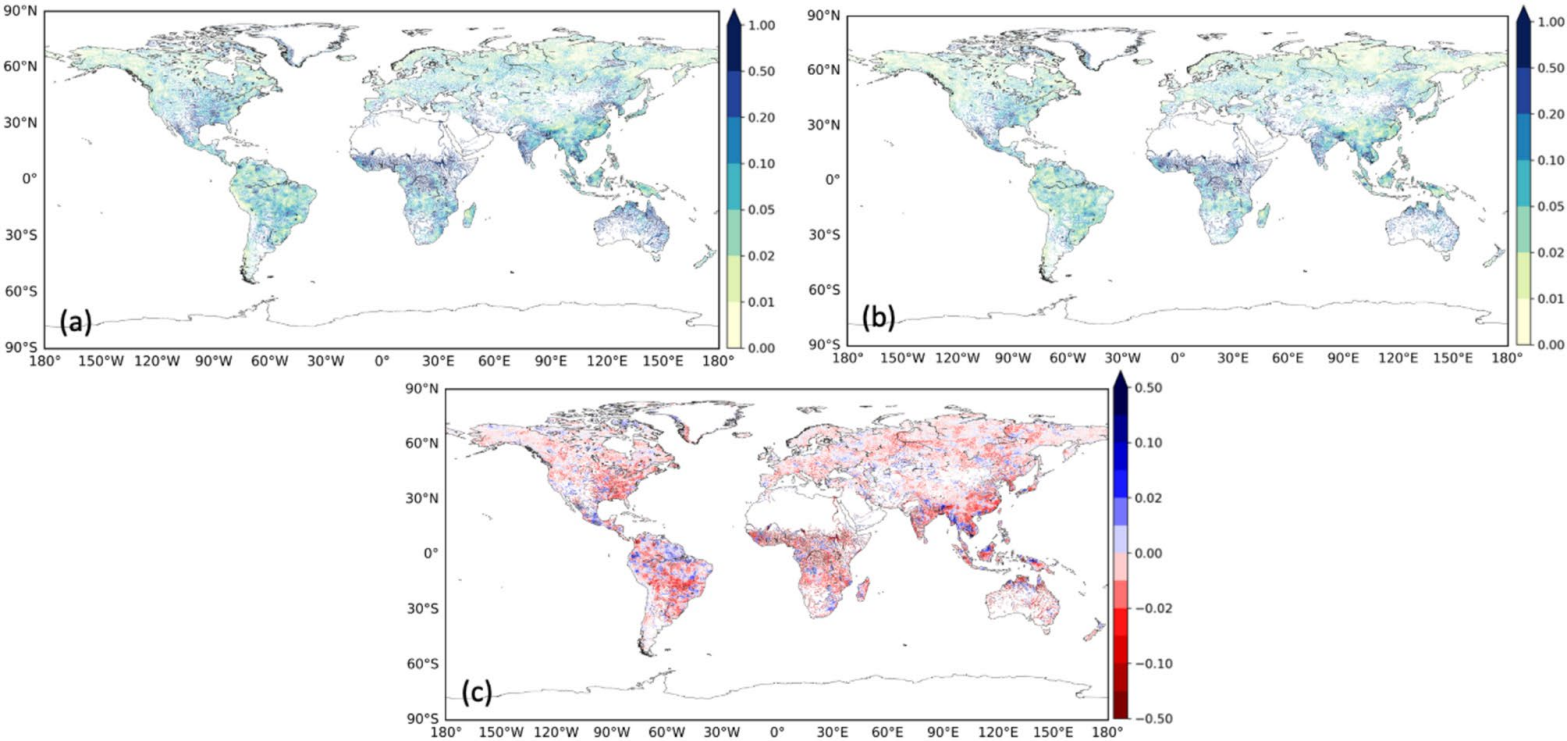
Unbiased variance in the flood change ratio from the historical climate to 2.0 °C warming. (**a**) among nine GCMs (only SSP5-RCP8.5) and (**b**) among nine GCMs integrating multiple SSP-RCP into a 90-year (or 60-year) time-series data pool at each GCM, performing an extreme value analysis and calculating unbiased variances. (**c**) Change in the variance from (**a**) to (**b**) are also shown.

The results of Fig. 2–5 show that the uncertainty in the flood projection could be reduced using our proposed method. The uncertainty in flood projection using our method was reduced in approximately 70% of the grid points compared to when the simulation was performed only with SSP5-RCP8.5 (Fig. 6). This pattern was particularly evident in grid points in which the unbiased variance among GCMs was larger than 0.1. In regions characterized by an initially significant unbiased variance among GCMs, the reduction increased to about 80% of the grid points, with significant decreases in the Mississippi River (USA) and extending from China to Southeast Asia. These results suggest that the uncertainty in flood projection can be reduced by our method. However, even after integrating multiple SSP-RCPs, regions with large unbiased variance among GCMs remained, indicating a large uncertainty, including differences in the physical parameters of climate models. The close-up views for Europe and Central Africa are shown in Supplementary Fig S7.

## Discussion

### Uncertainty in flood risk estimation

The variability (unbiased variance) among GCMs can be reduced by performing an extreme value analysis after integrating the annual maximum discharges of multiple SSP-RCPs for each GCM. While the main analysis of this study focused on evaluating the uncertainty in extreme river discharge projections under future climate conditions, it is important to assess how this uncertainty reduction translates to more societally relevant metrics such as flood risk. To this end, we utilized CaMa-Flood’s capability to generate floodplain inundation estimates to evaluate the impact on affected populations. This linkage between hydrological uncertainty and flood risk enables a more comprehensive understanding of the uncertainty reduction in climate projections.

The future climate inundation depth distribution (future flood hazard map) was constructed based on the change in future flood frequency at X°C warming, using the lookup method of^19^. Detailed explanations of the construction of a future flood hazard map and of the population used in this section are provided in Supplementary Text S1. We compared the use of our scenario-integration method to the conventional approach only using one scenario (SSP5-RCP8.5). Then, the affected population in each case at X°C warming was determined to estimate the magnitude of the reduction of uncertainty in future impact assessments that could be achieved using the proposed method.

Table 2 shows the change in the size of the affected population at X°C warming compared to the historical climate after applying the two approaches described above. The affected population was predicted to be 1.62 billion under the historical climate and increase by 0.048–0.083 billion (+ 2.9%- + 5.1%) in the case of 1.5–3 °C warming according to the future climate hazard map based on our proposed method. In addition, the proposed method reduced the variation (unbiased standard deviation) among the nine GCMs with respect to the affected population by 5–10% compared to only SSP5-RCP8.5, thus demonstrating its ability to reduce the uncertainty in future flood impact assessments.

**Table 2:** Change in the affected population at X°C warming vs. the historical climate according to the two approaches based on the lookup method. (unit: million). Note that the affected population was predicted to be 1.62 billion under the historical climate (1980–2014), the detailed explanations of the historical flood hazard map is provided in (Supplementary Text S1).

	At 1.5 °C warming		At 2 °C warming		At 3 °C warming	
	9GCM average	Unbiased Standard deviation among 9GCM	9GCM average	Unbiased Standard deviation among 9GCM	9GCM average	Unbiased Standard deviation among 9GCM
(1)Using only SSP5-RCP8.5	+ 47	39	+ 61	39	+ 80	34
(2) Using our proposed method	+ 48	37	+ 56	36	+ 83	31
Change ratio from (1) to (2)		− 5.1%		-7.9%		-9.7%

### Uncertainty in the scenario integration method

This study demonstrated that focusing on specific global warming levels—such as 1.5 or 2.0 °C—can reduce uncertainty in climate impact assessments by integrating outputs from multiple SSP-RCP scenarios. Our method assumes that climate projections are comparable across scenarios at the same warming level, an approach also used for mean climate variables (e.g.^26^). We extended this concept to extreme events, particularly flooding. However, the assumption that “climate impacts are comparable under the same warming level” may not hold uniformly across all climate variables. For example, Donnelly et al.^25^ showed that precipitation and runoff are broadly consistent among different SSP-RCPs at the same warming level. In contrast, Liu et al.^27^ found that permafrost degradation can differ significantly depending on the scenario, even at the same temperature level. Furthermore, for certain sectors—such as ecosystem management—risk assessments based solely on discrete warming levels may be insufficient. Overshoot scenarios, where global temperature temporarily exceeds critical thresholds, can cause irreversible impacts, highlighting the need for trajectory-based analyses in some applications.

Additional caution is warranted when applying this method to scenarios with markedly different socio-economic pathways. For instance, SSP3-RCP7.0 assumes lenient air quality policies, resulting in higher aerosol emissions and distinct precipitation patterns compared to other scenarios^8,28^. Such non-GHG factors can introduce inconsistencies in climate response across scenarios, potentially limiting the validity of integrated analysis.

Another source of methodological uncertainty is the number of ensemble members used to quantify climate variability. While our method showed that scenario integration can enhance the effective ensemble size, it remains unclear how many ensemble members are needed to sufficiently constrain uncertainty. As suggested by Shiogama et al.^8^, a more systematic investigation of ensemble size effects is required. Future studies using larger ensembles or perturbed initial condition experiments could better quantify the relationship between ensemble size and uncertainty reduction. Note that we also compared our proposed method—where SSP-RCP data are pooled into a 90-year time series prior to extreme value analysis—with an alternative approach that calculates extreme parameters independently for each 30-year scenario-specific segment. As shown in Supplementary Text S2 and Figures S8 and S9, our method yielded greater reductions in inter-model variance, supporting its effectiveness in enhancing robustness despite limited ensemble size.

### Other limitations

In this study, climate-induced changes in river discharge were analyzed using daily GCM runoff without applying bias correction. This choice was based on our prior work^19^, which demonstrated that relative changes in frequency or magnitude are more robust than absolute values when estimating future flood magnitude and inundation area. The “lookup method” used in that study links the projected change in flood frequency from raw GCM outputs with historical reanalysis-based inundation depth distributions. This approach avoids bias correction, which can itself introduce additional uncertainty, especially when future conditions diverge from historical reference periods. In addition, our analysis does not consider non-climatic impacts on river discharge such as human interventions (e.g., dam operations, irrigation) which can significantly influence runoff and flooding. Also, future sea level rise could increase the inundation area of low-lying regions^29^, but changes in water levels at the river mouth were not considered as boundary conditions in this study due to the uncertainty of the projections.

Furthermore, this study does not fully account for uncertainties in land surface hydrological processes, which are distinct from those in climate models. Structural assumptions in runoff generation, soil moisture storage, and routing schemes introduce independent uncertainty. These can be further amplified by the non-linear propagation of atmospheric model biases (Cloke & Pappenberger, 2009; Ferretti et al., 2020). Land use and land cover (LULC) changes—another major source of uncertainty—are only implicitly included through prescribed GCM boundary conditions. Future studies should consider multi-model hydrological ensembles and dynamic land surface representations to better capture these uncertainties.

Lastly, while CaMa-Flood includes sub-grid-scale channel representation and has been widely used for global flood assessments, it has limitations in resolving floodplain dynamics in urban or topographically complex regions. Local hydraulic features such as levees, drainage systems, and urban land use are not explicitly modeled. For such regions, local high-resolution hazard maps typically offer more precise information. However, where such maps are unavailable—particularly in developing countries—global models like CaMa-Flood still provide valuable first-order flood hazard assessments.

## Conclusions

This study investigated whether the uncertainty in future flood prediction due to small number of ensemble members could be reasonably reduced by merging multiple SSP-RCPs and extracting the periods with the same warming level under each SSP-RCP. The uncertainty due to small number of ensemble members is a one of the major sources of uncertainty in climate predictions. Projection uncertainty due to the above could be mitigated by increasing the effective number of ensemble members. However, only a limited number of large-ensemble experiments are available for each of the CMIP6 GCMs. Therefore, while increasing the ensemble size may be difficult, evaluating future projections of X°C warming by integrating multiple SSP-RCP with data at the time of that warming may increase the sample size. This study investigated whether the uncertainty in future flood prediction due to small number of ensemble members could be reasonably reduced by merging multiple SSP-RCPs and extracting those periods with the same warming level under each SSP-RCP.

A preliminary investigation of the similarity in flood projection at the same level of warming among SSP-RCP scenarios showed that at 2 °C warming the change ratio in the flood magnitude showed similar distributions for all SSP-RCPs. Moreover, the uncertainty due to the different SSP-RCPs (5–10%) was smaller than the difference in flood projection between 2 and 3 °C or between 3 and 4 °C (20–50%), which suggests that differences among SSP-RCPs as to future flood discharge change are relatively small. Accordingly, integrating multiple SSP-RCPs is an appropriate method for reducing the uncertainty due to small number of ensemble members in impact assessments at X°C warming.

The ability of our method to reduce the variability among GCMs regarding future flood changes was compared to the use of SSP5-RCP8.5 alone. The unbiased variance among GCMs in our method was reduced in about 70% of the grid points compared to when SSP5-RCP8.5 alone was applied. In regions characterized by an initially significant unbiased variance among GCMs, the reduction increased to about 80% of the grid points, with significant decreases in the Mississippi River (USA) and extending from China to Southeast Asia.

Finally, the proposed method was tested by creating future hazard maps based on the change in flood frequency in each GCM using the lookup method with only nine GCMs under the SSP5-RCP8.5 scenario vs. using the proposed method. Then the size of the affected population at X°C warming was calculated according to these two approaches. The results showed a reduction in the variation among GCMs of the affected population of 5–10%.

Based on the above results, our proposed method is very helpful for assessing climate change impacts because it could not only meet the growing need to evaluate impacts of specific warming levels but also reduce the uncertainty as to future flood impact assessment. Furthermore, the warming-level-based approach adopted in this study aligns more directly with international climate policy targets such as those set by the Paris Agreement (e.g., 1.5 and 2.0 °C warming goals). Unlike time- or scenario-based projections, which depend heavily on assumptions about socioeconomic development and emissions pathways, warming-level approaches enable more consistent comparison of physical climate responses across scenarios. This approach is particularly relevant for risk assessments and adaptation planning because it focuses on the impacts associated with specific levels of global warming, irrespective of the pathway taken to reach them. Therefore, it facilitates actionable insights for policymakers by linking projected hazards—such as floods—to clearly defined policy-relevant temperature thresholds. Thus, the proposed method is expected to be commonly used as a method to reduce the uncertainty of small number of ensemble members regarding future projections in CMIP6 and to provide a more accurate and helpful estimates of the impacts of climate change.

## Supplementary Information

Supplementary Information.

## Data Availability

The topography data MERIT DEM are available from http://hydro.iis.u-tokyo.ac.jp/~yamadai/MERIT_DEM/index.html (last access: 17 August 2023)^30^. The CMIP6 data are available from the Earth System Grid Federation (ESGF) data platform (https://esgf-node.llnl.gov/search/cmip6/, accessed on 17 August 2023).

## Code availability

The global hydrodynamic model CaMa-Flood (v4.10) is available from (https://zenodo.org/records/7597409)^18^.
