# Supplementary material for: Reduction of the uncertainty of flood projection under a future climate by focusing on similarities among multiple SSP-RCP scenarios
Source: Sci Rep. 2025 Sep 22;15:31614. doi: 10.1038/s41598-025-16327-4 (PMC12454635; doi:10.1038/s41598-025-16327-4)
Supplement: Supplementary file 1 — Supplementary Information. [file 41598_2025_16327_MOESM1_ESM.docx]

**Supplementary Materials for**

**Reduction of the uncertainty of flood projection under future climate by focusing on similarities among multiple SSP-RCP scenarios:**

*Yuki Kimura^1 2^, Yukiko Hirabayashi^3^, Dai Yamazaki^2^

^1^Data Analytics Department, MS&AD InterRisk Research & Consulting, Inc. , 2-105, Kanda Awajicho, Chiyoda-ku, Tokyo 101-0063, Japan

^2^Institute of Industrial Science, The University of Tokyo, 4-6-1 Komaba, Meguro-ku, Tokyo, 153-8505, Japan

^3^Department of Civil Engineering, Shibaura Institute of Technology, 3-7-5 Toyosu, Koto-ku, Tokyo, 135-8548, Japan

**Correspondence to*: Y. Kimura (yu1590yu@gmail.com)

**Supplementary material comprises:**

Table S1. Time to reach SWLs from the preindustrial global mean temperature under SSP-RCP scenarios.

Table S2. GCM ensemble lists

Table S3. Quantitative evaluation of similarities between different SSP-RCPs. “b” is the slope of the fitted line using principal component regression.

Fig. S1. Spatial distribution of the flood change ratio from the historical climate to 2°C warming. Same as the Fig.2 but for EC-Earth3

Fig. S2. Spatial distribution of the flood change ratio from the historical climate to 2°C warming. Same as the Fig.2 but for ACCESS-CM2

Fig S3. Spatial distribution of the flood change ratio. Close up view of Fig.2 for Europe and Central Africa.

Fig. S4. Comparison of the flood change ratio among two simulations. Same as the Fig.3 but for EC-Earth3

Fig. S5. Comparison of the flood change ratio among two simulations. Same as the Fig.3 but for ACCESS-CM2

Fig S6. Spatial distribution of the standard deviation of the flood change ratio. Close up view of Fig 4 for Europe and Central Africa.

Fig S7. Unbiased variance in the flood change ratio. Close up view of Fig 6 for Europe and Central Africa.

Fig. S8. Unbiased variance in the flood change ratio from the historical climate to 2.0°C warming . Same as the Fig.6 but (b) is an alternative method

Fig. S9. Histogram showing the change in unbiased variance nine GCMs by different methods.

Unbiased variance among GCMs at 3.0°C warming under SSP5-RCP8.5.

Text S1: Method used to construct a future flood hazard map at X°C warming and the population data used in maintext Section 4.1

Text S2: Alternative ensemble treatment method for integrating SSP-RCPs

**Table S1.Time to reach SWLs from the preindustrial (1850-1900) global mean temperature under SSP-RCP scenarios. *For these GCMs, multiple ensembles were used**

|  |  | Specific warming levels | | | |
| --- | --- | --- | --- | --- | --- |
| GCM | SSP-RCP | 1.5°C | 2.0°C | 3.0°C | 4.0°C |
| ACCESS-CM2(*) | 1-2.6 | 2028 | 2043 | - | - |
| ACCESS-CM2(*) | 2-4.5 | 2028 | 2041 | 2071 | - |
| ACCESS-CM2(*) | 5-8.5 | 2026 | 2038 | 2056 | 2071 |
| EC-Earth3(*) | 1-2.6 | 2023 | 2050 | - | - |
| EC-Earth3(*) | 2-4.5 | 2024 | 2044 | 2085 | - |
| EC-Earth3(*) | 5-8.5 | 2024 | 2036 | 2057 | 2074 |
| GFDL-CM4 | 2-4.5 | 2032 | 2050 | - | - |
| GFDL-CM4 | 5-8.5 | 2030 | 2041 | 2061 | 2079 |
| INM-CM5-0 | 1-2.6 | 2042 | - | - | - |
| INM-CM5-0 | 2-4.5 | 2038 | 2072 | - | - |
| INM-CM5-0 | 5-8.5 | 2031 | 2046 | 2074 | - |
| IPSL-CM6A-LR(*) | 1-2.6 | 2019 | 2038 | - | - |
| IPSL-CM6A-LR(*) | 2-4.5 | 2019 | 2034 | 2064 | - |
| IPSL-CM6A-LR(*) | 5-8.5 | 2018 | 2033 | 2051 | 2066 |
| MIROC6 | 1-2.6 | 2075 | - | - | - |
| MIROC6 | 2-4.5 | 2046 | 2076 | - | - |
| MIROC6 | 5-8.5 | 2039 | 2053 | 2076 | - |
| MPI-ESM1-2-HR | 1-2.6 | 2042 | - | - | - |
| MPI-ESM1-2-HR | 2-4.5 | 2037 | 2061 | - | - |
| MPI-ESM1-2-HR | 5-8.5 | 2033 | 2049 | 2074 | - |
| MRI-ESM2-0 | 1-2.6 | 2032 | - | - | - |
| MRI-ESM2-0 | 2-4.5 | 2032 | 2049 | - | - |
| MRI-ESM2-0 | 5-8.5 | 2026 | 2039 | 2064 | 2084 |
| NorESM2-MM | 2-4.5 | 2049 | 2081 | - | - |
| NorESM2-MM | 5-8.5 | 2041 | 2054 | 2076 | - |

**Table S2. GCM ensemble lists**

| GCM | Ensemble |
| --- | --- |
| ACCESS-CM2 | r1i1p1f1, r4i1p1f1, r5i1p1f1 |
| EC-Earth3 | r1i1p1f1, r4i1p1f1, r5i1p1f1 |
| IPSL-CM6A-LR | r1i1p1f1, r2i1p1f1, r3i1p1f1 |

**Table S3. Quantitative evaluation of similarities between different SSP-RCPs. “b” is the slope of the fitted line using principal component regression.**

|  | Value of “b”, which is slope of the fitted line | | |
| --- | --- | --- | --- |
|  | IPSL-CM6A-LR0 | EC-Earth3 | ACCESS-CM2 |
| (a) at2.0°C warming_ **SSP1-RCP2.6_SSP5-RCP8.5** | 1.02 | 1.01 | 0.94 |
| (b) at2.0°C warming_ **SSP2-RCP4.5_SSP5-RCP8.5** | 1.01 | 0.91 | 1.01 |
| (c) SSP1-RCP2.6_ +**1.5°C_+2.0°C warming** | 1.5 | 1.33 | 1.17 |
| (d) SSP2-RCP4.5_ +**1.5°C_+2.0°C warming** | 1.45 | 1.3 | 1.2 |
| (e) SSP5-RCP8.5_ +**2.0°C_+3.0°C warming** | 1.29 | 1.54 | 1.38 |
| (f) SSP5-RCP8.5_ +**3.0°C_+4.0°C warming** | 1.28 | 1.35 | 1.28 |


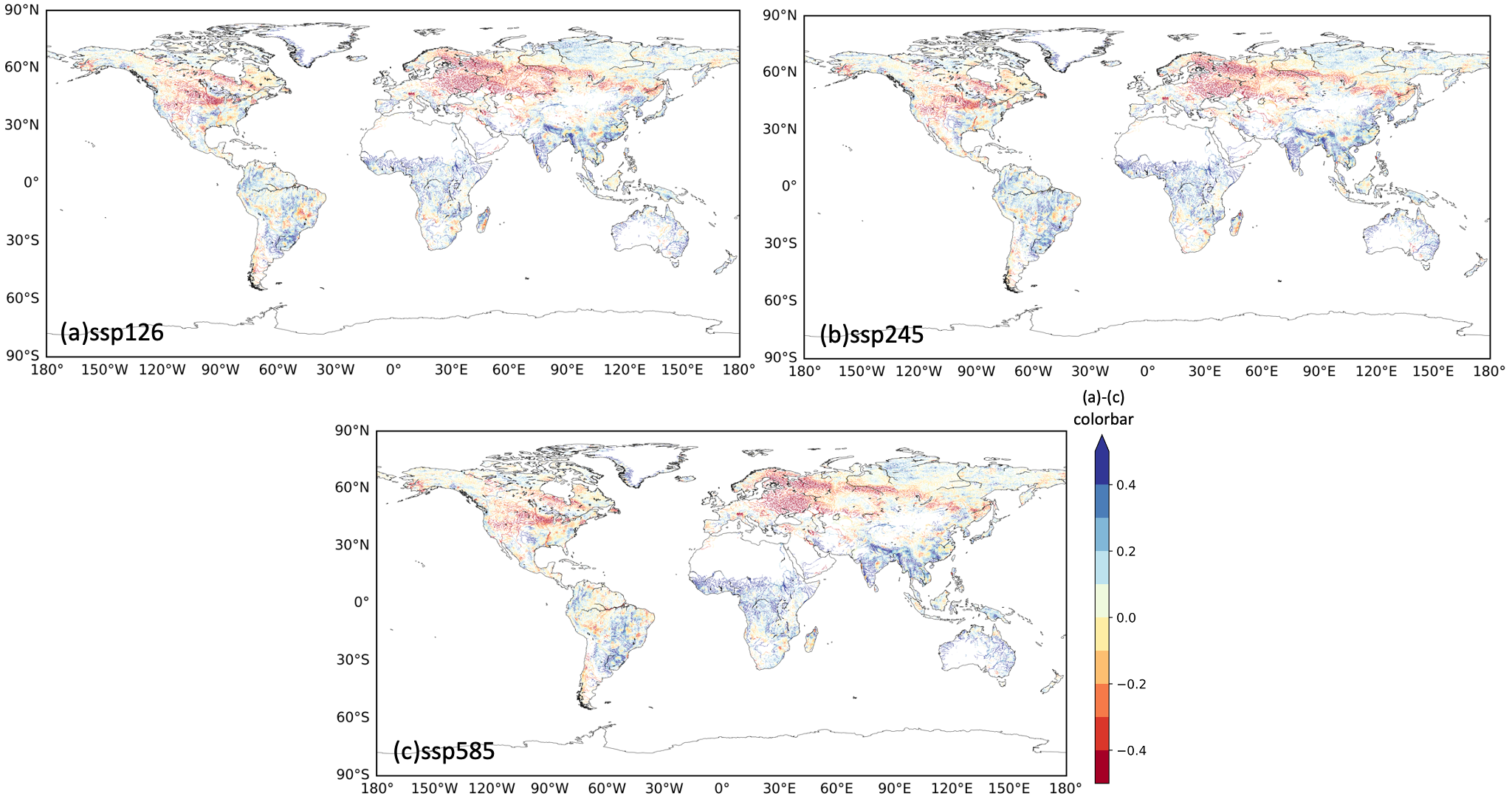


**Figure S1. Spatial distribution of the flood change ratio.** Same as the Fig. 2 but for EC-Earth3


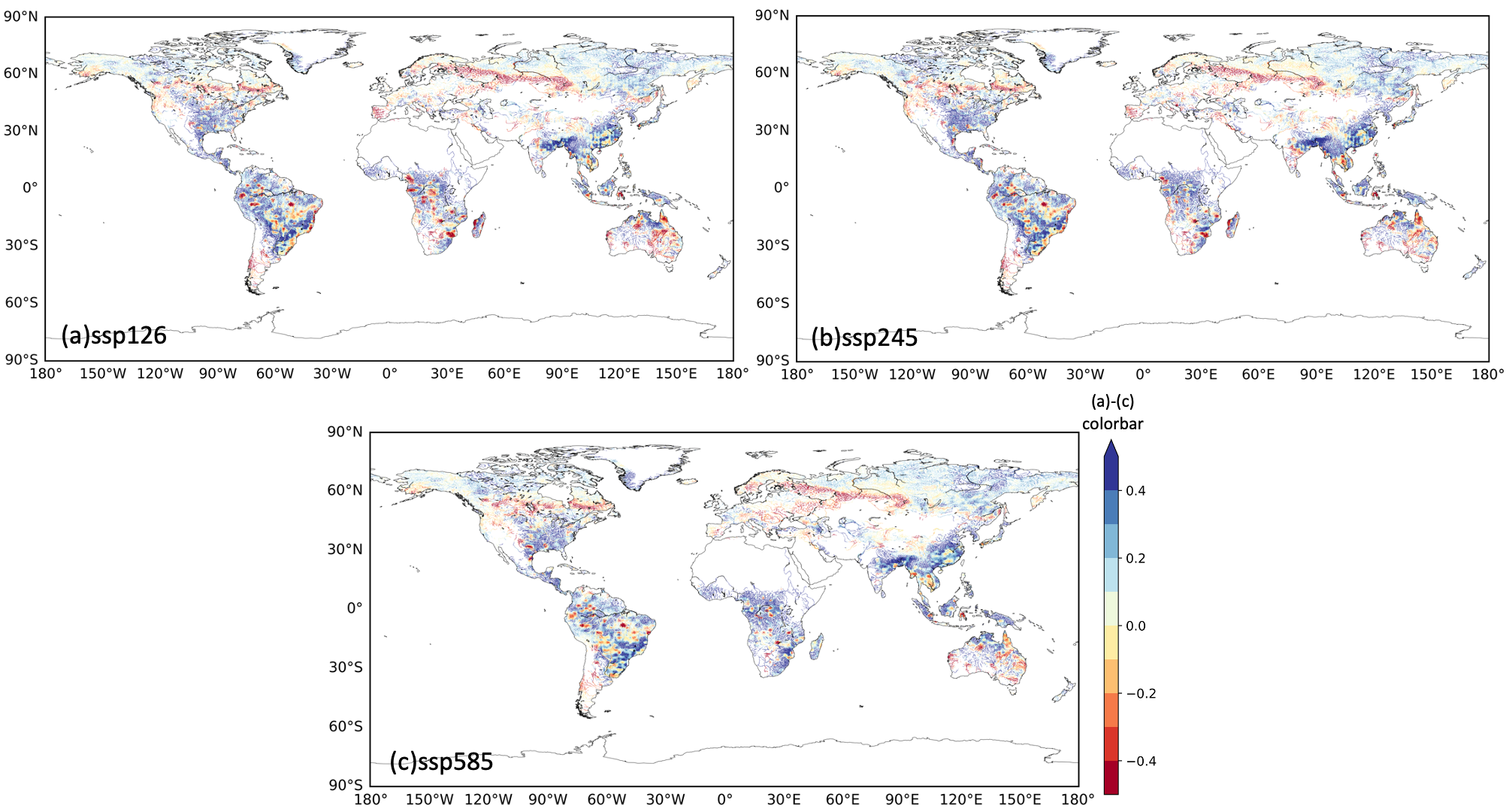


**Figure S2. Spatial distribution of the flood change ratio.** Same as the Fig. 2 but for ACCESS-CM2


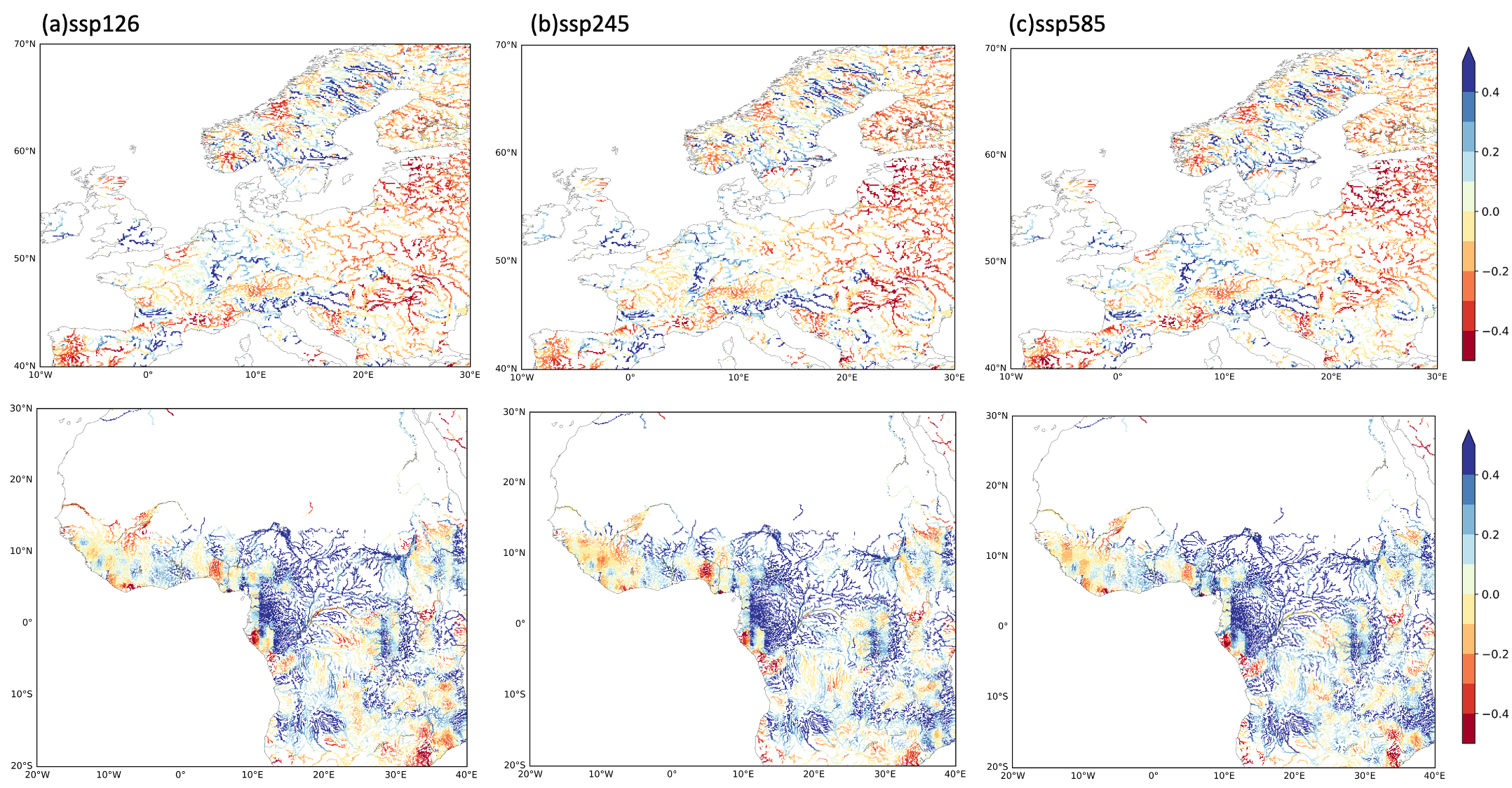


**Figure S3. Spatial distribution of the flood change ratio.** Close up view of Fig.2 for Europe and Central Africa.

**
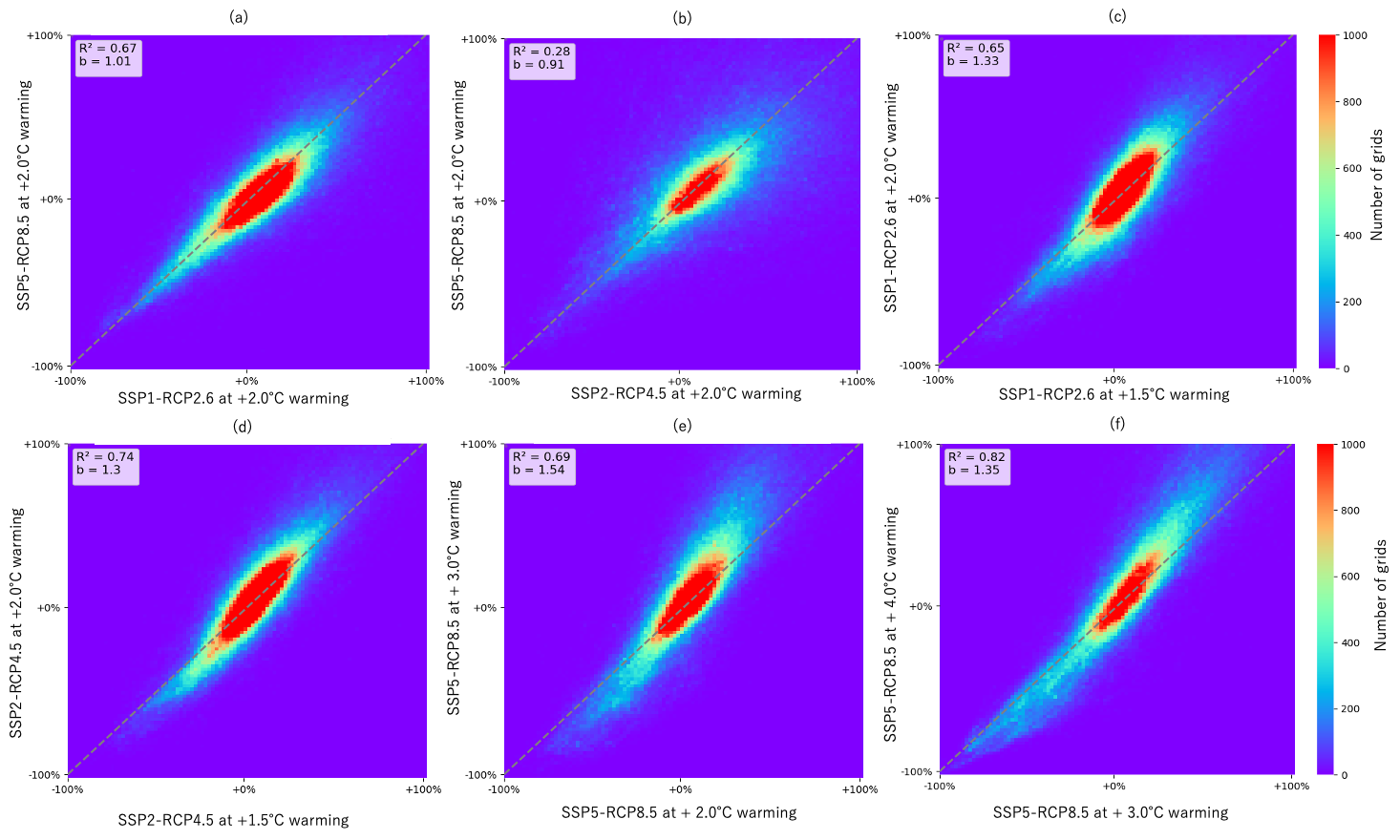
**

**Figure S4. Comparison of the flood change ratio among two simulations.** Same as the Fig. 3 but for EC-Earth3.

**
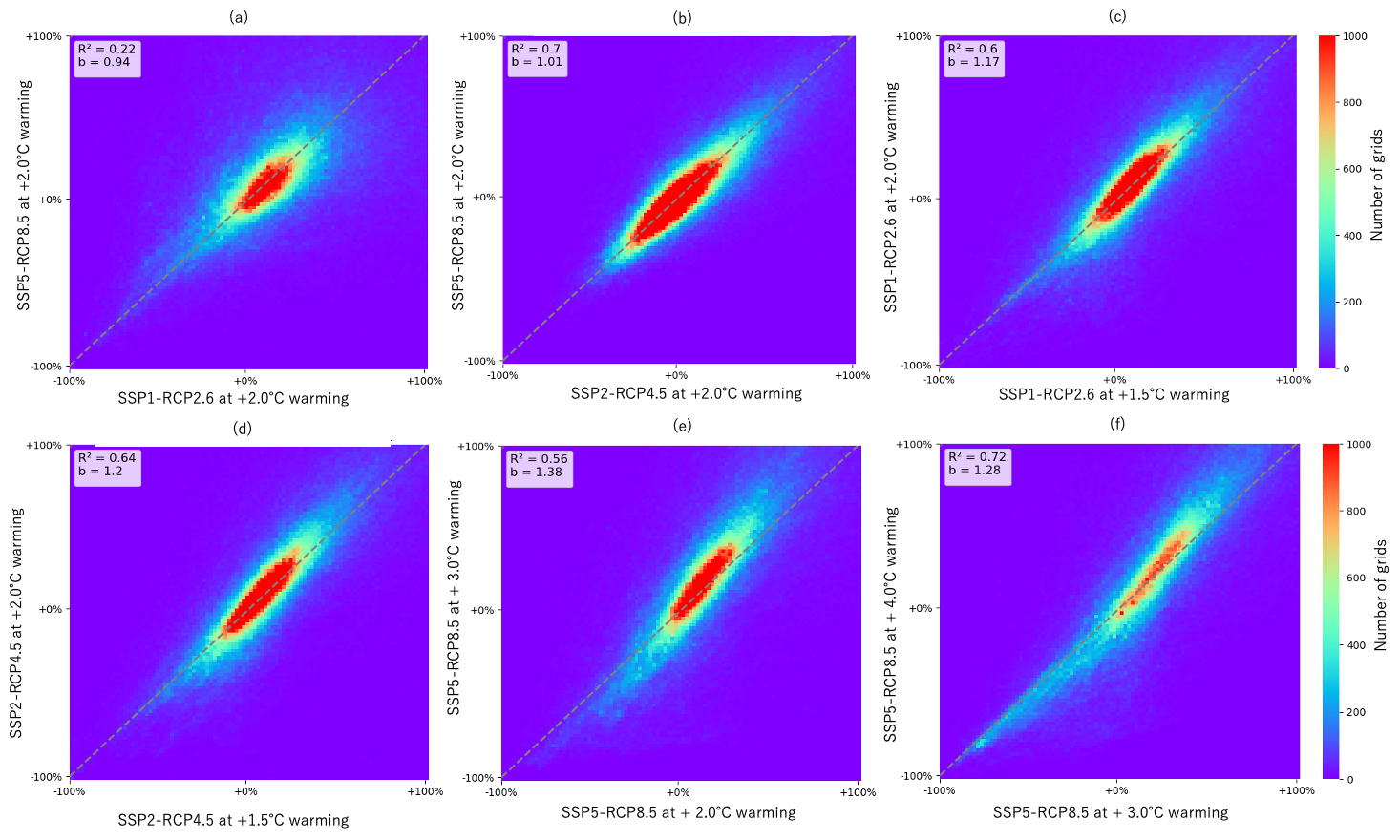
**

**Figure S5. Comparison of the flood change ratio among two simulations.** Same as the Fig. 3 but for ACCESS-CM2


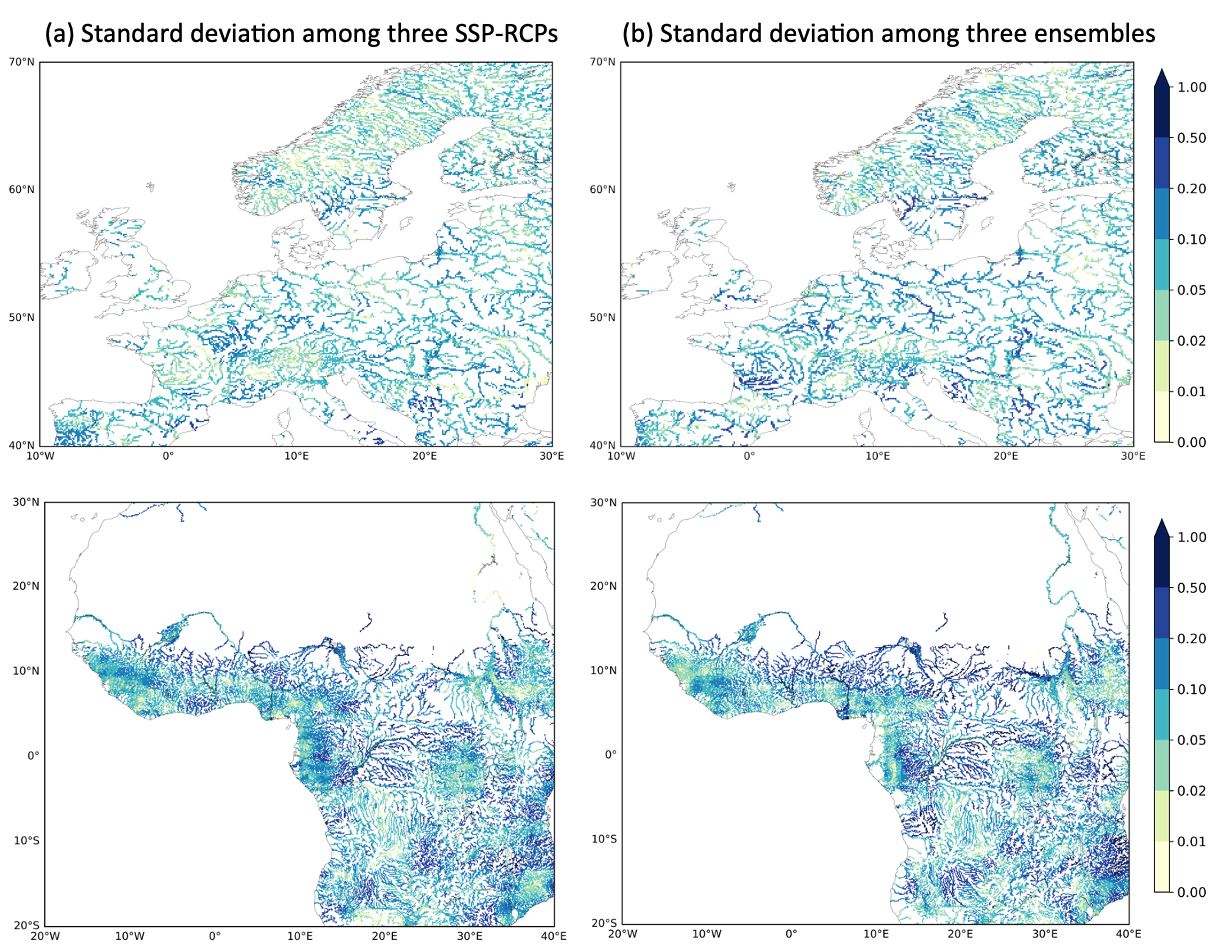


**Figure S6. Spatial distribution of the standard deviation of the flood change ratio.** Close up view of Fig 4 for Europe and Central Africa.


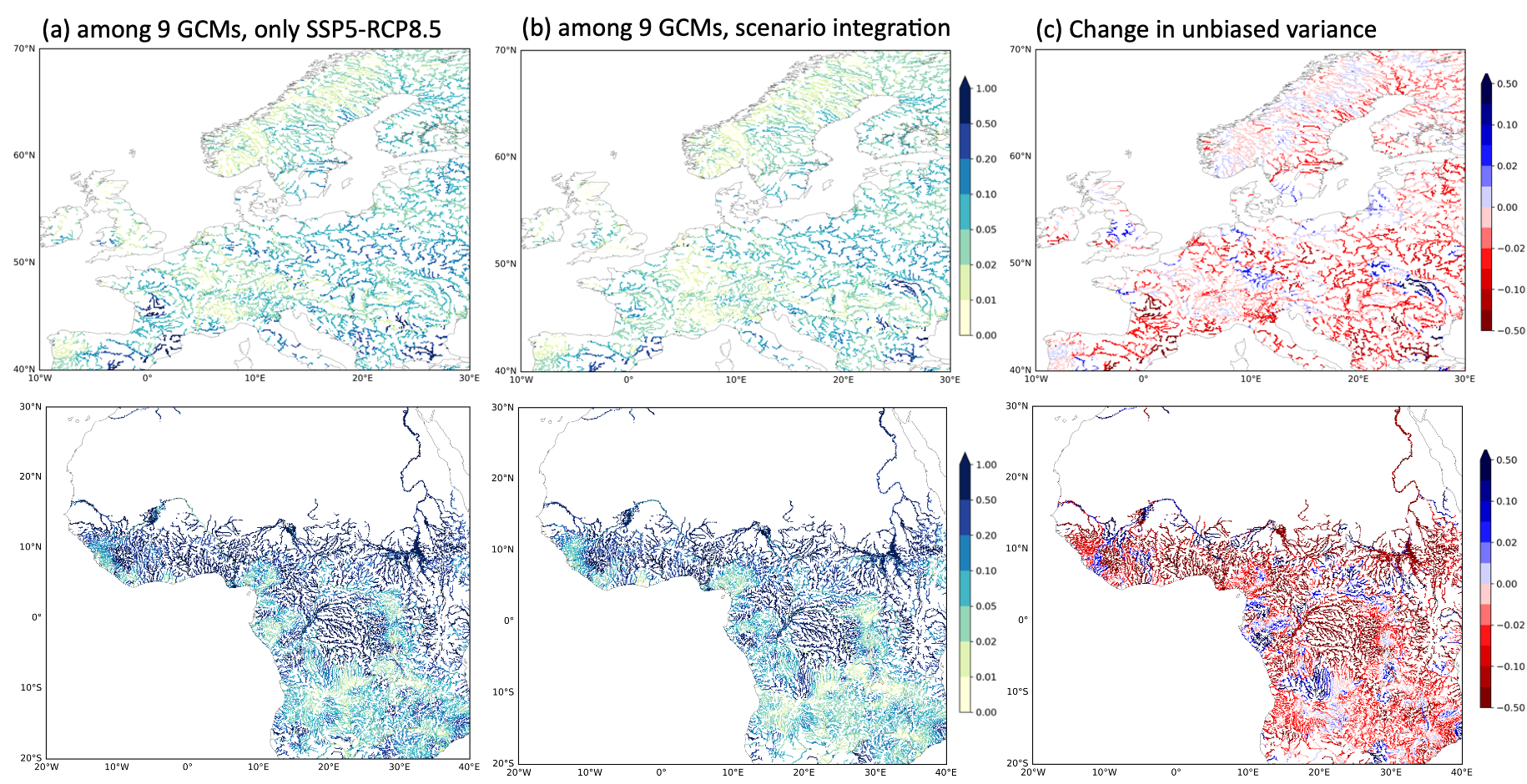


**Figure S7. Unbiased variance in the flood change ratio.** Close up view of Fig 6 for Europe and Central Africa.


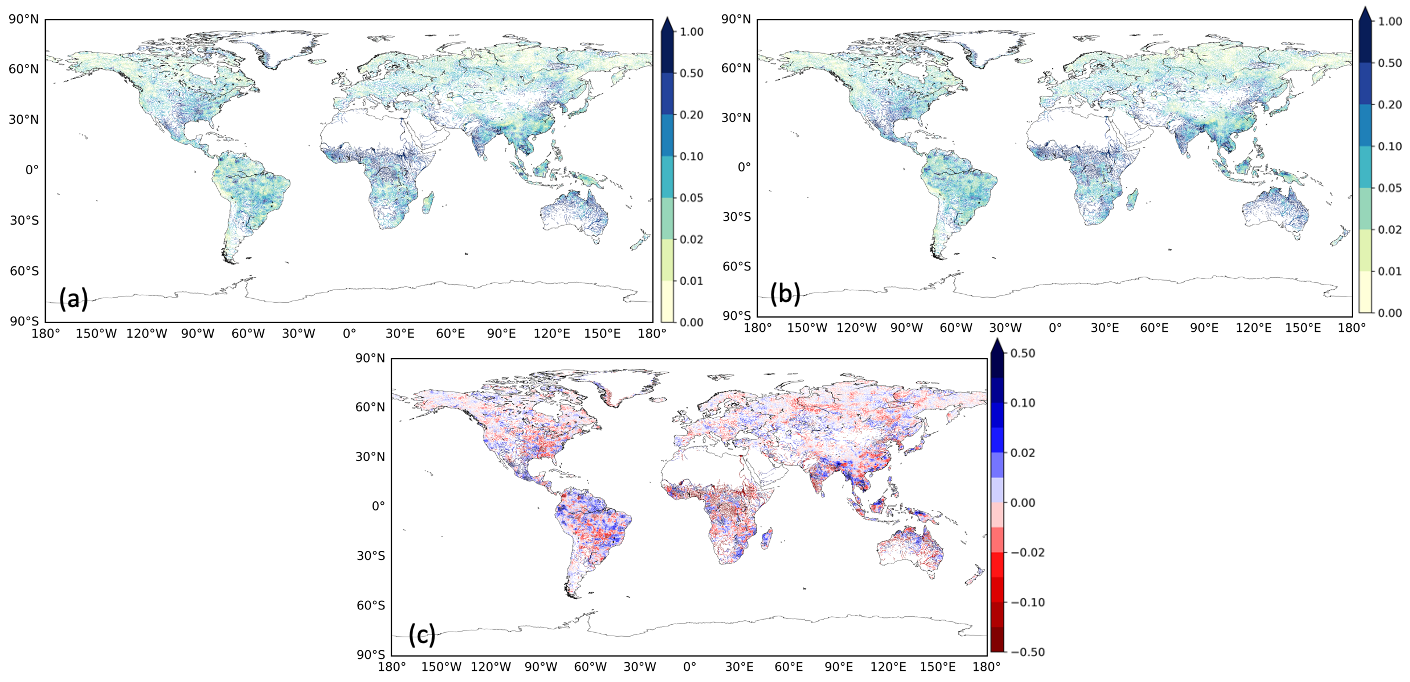


**Figure S8. Unbiased variance in the flood change ratio from the historical climate to 2.0°C warming.** Same as the Fig.5 but (b) is an alternative method (for a detailed explanation, see Text S1)


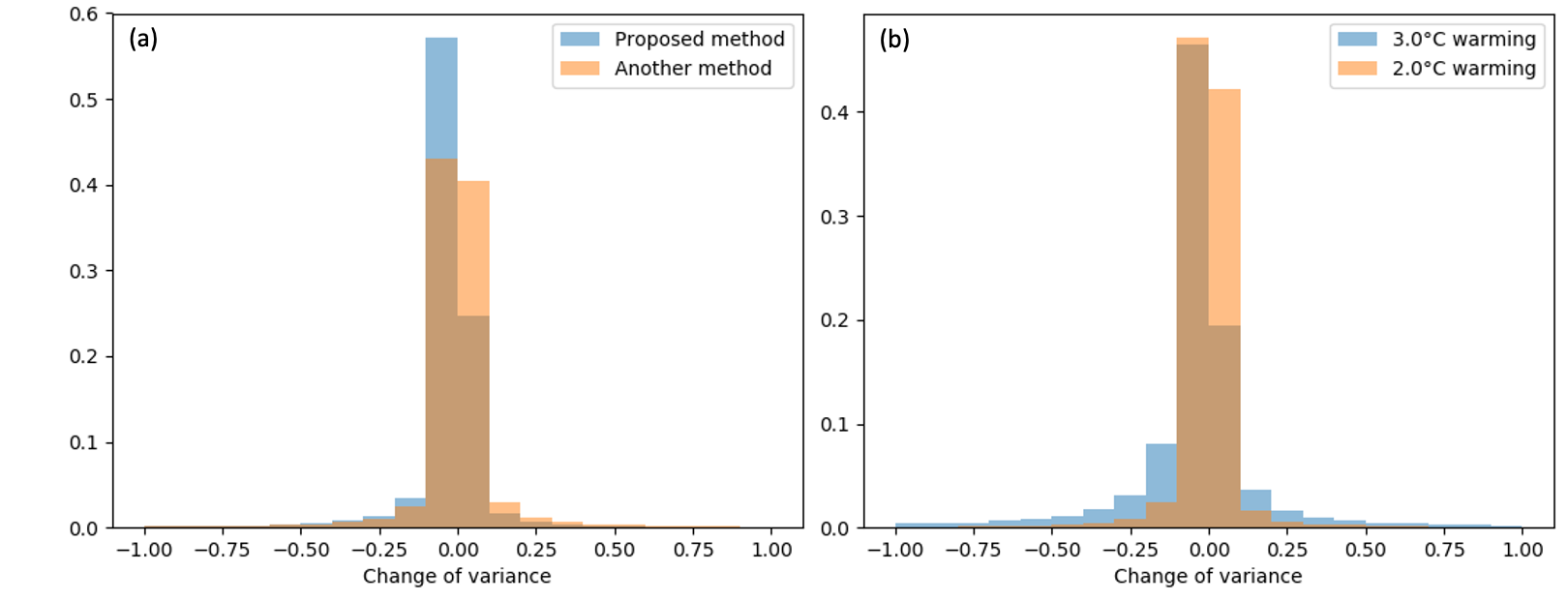


Figure S9: Histogram showing the change in unbiased variance nine GCMs by different methods. The change by using scenario integration compared to only using SSP5-RCP8.5. Change by the proposed method is blue, while change by the individual 30-year approach is in orange. Note that the unbiased variance among the nine GCMs is defined based on 100-year RP discharge change ratio from the historical climate.

# Text S1: Method used to construct a future flood hazard map at X°C warming and the population used in maintext Section 4.1

The future climate inundation depth distribution (future flood hazard map) was constructed based on the change in future flood frequency at X°C warming using the lookup method of Kimura et al., 2023. The 100-year RP inundation depth distribution was used to calculate the flood risk (see maintext Section 4.1). The lookup method uses GCM simulation results without bias correction to calculate change in future flood frequency and then constructs a future climatic inundation depth distribution for the target RP based on each inundation depth distribution determined in the RP reanalysis. VIC-Bias Corrected (Yang et al., 2021) was used as the reanalysis data and a CaMa-Flood river inundation simulation (6 min resolution) was run to calculate the inundation depths for each RP under the historical climate (1980-2014). 100-year RP inundation depth based on VIC-Bias Corrected was used to calculate affected population under the historical climate.

Our proposed method, which reduces uncertainties due to GCM climatic sensitivity and GCM climate internal variability, was used to calculate the future change in flood frequency according to the lookup method. The change at X°C warming, which refers to the RP under the historical climate corresponding to the 100-year RP water levels at X°C warming, was calculated for each GCM. In our proposed method, we used multiple SSP-RCPs at X°C warming through this procedure to perform extreme value analysis. Regarding extreme value analysis, with the L-Moments method (Hosking, 2015), we fitted the Gumbel distribution to the annual maximum river level of multiple SSP-RCPs, picking up 30-year used for calculating specific warming levels (30-year sample including 15 years before and 14 years after the SWL year, please see Supplementary Tables S1) from each SSP-RCP.

The value of the each GCM was set as the change in future flood frequency and used in the lookup method to calculate 100-year RP river level of the future climate in each GCM. Downscaling was performed under the assumption that the water surface elevation is uniform within each 6 min unit catchment. The floodplain water depth was thus defined as the point when the terrain elevation of a 30-arcsec pixel was lower than the water surface elevation. See the methods of Kimura et al 2023 and Yamazaki et al, 2011 for details. The future hazard maps created based on a flood change of each GCM were used to calculate the affected population and calculate the average and unbiased standard deviation of the 9 GCMs regarding the affected population.

The impacts of flood exposure on a population were estimated based on the inundation map and the 2020 population density map (Gridded Population of the World. GPW) (CIESIN, 2018). Because the map is in 30 arcsec resolution, a 30 arcsec inundation map was created by downscaling the river water levels calculated using the lookup method. As discussed by Zhou et al., 2021, the spatial resolution of flood hazard maps is a particularly important factor in determining their value in impact assessment. Smith et al., 2019 evaluated the exposure of residents to a 100-year RP flood in 18 developing countries, including when the spatial resolution of flood hazard maps was reduced from 90 m to 900 m; the exposures for different population areas increased by 51-94%. Despite the uncertainty in the choice of the spatial resolution of the flood hazard maps, 30 arcsec instead of 3 arcsec resolution was chosen for a global comparison of X°C warming levels because 30 arcsec was computationally convenient.

# Text S2: Alternative ensemble treatment method for integrating SSP-RCPs

In our proposed method, multiple SSP-RCPs are first integrated into a 90-year (or 60-year) time-series data set at each GCM, and then extreme parameters are calculated to reduce uncertainties in flood hazard projection. The advantages of our method are that the sample size is increased for extreme value estimation and the uncertainty due to small number of ensemble members is reduced. However, it is possible that the order of ensemble integration and extreme parameter estimation can be swapped. In this section, we present another method for multiple SSP-RCP integration, in which extreme parameters are first calculated individually from a 30-year sample for each SSP-RCP, and then the unbiased variance in the flood change is calculated (i.e. individual 30-year approach).

The method based on individual 30-year approach is described in detail as follows. 1) The GCMs and SSP-RCPs that reached 2.0°C from nine GCMs and three SSP-RCPs (Supplement Table 1) resulted in 21 GCM-SSP-RCP for used in this study. (2) For each GCM-SSP-RCP, the annual maximum discharge for 30 years from T-15 to T+14 (T is the year in which 2°C is reached in Supplementary Table 1) was calculated and an extreme value analysis was performed to calculate the 100-year RP discharge under the future climate. (3) An extreme value analysis was also performed for the 1980-2014 annual maximum discharge for the historical climate of each GCM to calculate the 100-year RP discharge. Then, the 100-year RP discharge change ratio at 2°C warming for each GCM-SSP-RCP was calculated using Equation 1. (4) The unbiased variance of the 100-year RP discharge change ratio among the 21 GCM-SSP-RCP was calculated at each grid point.

We evaluated the impact of SSP-RCP integration by the two different methods (i.e. proposed and individual 30-year approaches), by comparing how unbiased variance in future flood change was reduced by the two methods compared to the case without scenario integration (i.e. only SSP5-RCP8.5 is used). The benefit by the individual 30-year method was limited, showing uncertainty reduction only in approximately 50% of the grid points compared to the proposed method (Supplementary Fig. S5, S6). There are a couple of possible reasons for this. It may be due to the impact of uncertainty in extreme value analysis due to a small sample size; i.e., a 30-year sample of annual maximum daily discharge may not be sufficient for extreme value analysis in the evaluation of flood with a 100-year return period, such that uncertainty remains. In addition, uncertainty surrounds projections for low warming and near-term scenarios (e.g., 2°C warming) (Maher et al., 2021), with the possibility of substantial uncertainty due to internal climate variability. Increasing the sample size without reducing the uncertainty of each GCM-SSP may not reduce the variability among GCMs. Our results demonstrate the importance of integrating the SSP-RCP prior to the extreme value analysis to reduce the uncertainty due to small number of ensemble members

# References in Supporting Information

Center for International Earth Science Information Network (CIESIN), Columbia University (2018), Documentation for the Gridded Population of the World, Version 4 (GPWv4), Revision 11 Data Sets, NASA Socioeconomic Data and Applications Center (SEDAC), Palisades, NY, <https://doi.org/10.7927/H45Q4T5F>.

Hosking, J. R. M.(2015), L-Moments, in: Wiley StatsRef: Statistics Reference Online, John Wiley & Sons, Ltd., Hoboken, USA, 1–8, <https://doi.org/10.1002/9781118445112.stat00570.pub2>.

Kimura, Y., Hirabayashi, Y., Kita, Y., Zhou, X., and Yamazaki, D. (2023), Methodology for constructing a flood-hazard map for a future climate. Hydrology and Earth System Sciences, 27(8), 1627-1644., <https://doi.org/10.5194/hess-27-1627-2023>

Maher, N., Milinski, S., and Ludwig, R. (2021), Large ensemble climate model simulations: introduction, overview, and future prospects for utilising multiple types of large ensemble. Earth System Dynamics, 12(2), 401-418., <https://doi.org/10.5194/esd-12-401-2021>

Smith, A., Bates, P. D., Wing, O., Sampson, C., Quinn, N., and Neal, J.(2019), New estimates of flood exposure in developing countries using high-resolution population data, Nat. Commun., 10, 1–7, <https://doi.org/10.1038/s41467-019-09282-y>.

Yamazaki, D., Kanae, S., Kim, H., and Oki, T. (2011), A physically based description of floodplain inundation dynamics in a global river routing model. Water Resources Research, 47.4, <https://doi.org/10.1029/2010WR009726>.

Yang, Y., Pan, M., Lin, P., Beck, H. E., Zeng, Z., Yamazaki, D., David, C.H., Lu, H. Yang, K., Hong, Yang. And Wood, E, F.(2021), Global Reach-Level 3-Hourly River Flood Reanalysis (1980–2019). Bulletin of the American Meteorological Society, 102.11, E2086-E2105, <https://doi.org/10.1175/BAMS-D-20-0057.1>.

Zhou, X., Ma, W., Echizenya, W., and Yamazaki, D.(2021), The uncertainty of flood frequency analyses in hydrodynamic model simulations. Natural Hazards and Earth System Sciences, 21(3), 1071-1085, <https://doi.org/10.5194/nhess-21-1071-2021>

# Data availability

VIC-Bias-Corrected runoff are available from https://www.reachhydro.org/home/records/grfr (Yang et al., 2021).

The English in this document has been checked by at least two professional editors, both native speakers of English. For a certificate, please see: http://www.textcheck.com/certificate/ZwVi4C
